# Editorial Notes

**Published:** 1883-06

**Authors:** 


### EDITORIAL NOTES.

Dr. J. D. Hill and family sailed for Europe last month, intending to remain abroad a year.

Another of the editors of the Journal, Dr. P. W. Van Peyma, sailed for Europe last week, intending to spend some months in study and recreation in Germany and Northern Europe. We are promised an occasional contribution from him, and we therefore hope that the series of letters which Dr. Davidson has supplied will not terminate with his return, but will be continued and supplemented by Dr. Van Peyma.

Dr. Lothrop's accident and the absence of Drs. Davidson and Van Peyma left the Journal without an editor. We would express our thanks to Drs. Howe, Peterson, Tremaine, and others who volunteered their kind assistance in the issue of the Journal for this month. The happy return of Dr. Davidson from Europe last week, however, made it unnecessary to accept their kind services, the offer of which is, however, not the less appreciated.

Dr. Thomas Lothrop met with a severe accident three weeks ago, which has entirely incapacitated him for business. In making his professional rounds, while driving on Delaware avenue the front axle of his buggy suddenly broke and the Doctor was thrown to the pavement, receiving severe contusions on his head and face, and was picked up insensible. Under the skillful treatment of Dr. Tremaine he has rapidly improved, and we hope will soon be at work again. It is to Dr. Lothrop's literary ability and large medical experience that the Journal owes its continued improvement and growing favor, and our readers will rejoice with us that our able confrere escaped any more serious injury.

